# *BRAF* and MEK Targeted Therapies in Pediatric Central Nervous System Tumors

**DOI:** 10.3390/cancers14174264

**Published:** 2022-08-31

**Authors:** Dario Talloa, Silvia Triarico, Pierpaolo Agresti, Stefano Mastrangelo, Giorgio Attinà, Alberto Romano, Palma Maurizi, Antonio Ruggiero

**Affiliations:** 1 Scuola di Specializzazione in Pediatria, Università Cattolica del Sacro Cuore, Largo F.sco Vito 1, 00168 Rome, Italy; 2 UOSD di Oncologia Pediatrica, Dipartimento di Scienze della Salute della Donna, del Bambino e di Sanità Pubblica, Fondazione Policlinico Universitario A. Gemelli IRCCS, Argo A. Gemelli 8, 00168 Rome, Italy; 3 Dipartimento di Scienze della Vita e Sanità Pubblica, Università Cattolica del Sacro Cuore, Largo F.sco Vito 1, 00168 Rome, Italy

**Keywords:** central nervous system (CNS) tumors, *BRAF*, MEK, children, NGS, targeted therapies

## Abstract

**Simple Summary:**

This review is divided into two parts. The first analyzes the mechanisms of two important cellular pathways that are involved in tumoral proliferation, differentiation, migration, and angiogenesis: RAS/RAF/MEK/MAPK and PI3K/AKT/mTOR. The second part focuses on the currently available experience regarding targeted therapies against the mitogen-activated protein kinase (MAPK) pathway in pediatric CNS tumors, with the hope of offering a practical guide for consultation.

**Abstract:**

*BRAF* is a component of the MAPK and PI3K/AKT/mTOR pathways that play a crucial role in cellular proliferation, differentiation, migration, and angiogenesis. Pediatric central nervous system tumors very often show mutations of the MAPK pathway, as demonstrated by next-generation sequencing (NGS), which now has an increasing role in cancer diagnostics. The MAPK mutated pathway in pediatric CNS tumors is the target of numerous drugs, approved or under investigation in ongoing clinical trials. In this review, we describe the main aspects of MAPK and PI3K/AKT/mTOR signaling pathways, with a focus on the alterations commonly involved in tumorigenesis. Furthermore, we reported the main available data about current *BRAF* and MEK targeted therapies used in pediatric low-grade gliomas (pLLGs), pediatric high-grade gliomas (pHGGs), and other CNS tumors that often present *BRAF* or MEK mutations. Further molecular stratification and clinical trial design are required for the treatment of pediatric CNS tumors with *BRAF* and MEK inhibitors.

## 1. Introduction

Central nervous system (CNS) tumors are the most common solid tumor in children and their management represents a challenge because of the sanctuary constituted by the blood-brain barrier (BBB) which protects tumors of the CNS from exposure to many active drugs [1].

Currently various genomic analysis techniques are used in the development of targeted therapies, and one of these is next-generation sequencing (NGS). NGS is a technology that is used to detect the nucleotide sequence of entire genomes or target regions of DNA or RNA [2]. NGS may discover novel mutations and entirely study cancer genomes, as shown in several large-scale cancer genome projects, such as a dedicated pediatric cancer genome project [3].

*BRAF* is an important component of the MAPK signaling pathway involved in cellular proliferation, differentiation, migration, and angiogenesis [4]. The regulation of *BRAF* is complex and subject to multiple areas of control, with numerous cross-talk between this route and other cellular signaling cascades, one of which is PI3K/AKT/mTOR. *BRAF* mutations have been found in many cancers, hence their relevance in the field of pharmaceutical research for the purpose of developing new targeted drugs [5].

In the field of pediatric CNS tumors, important progress has been made in molecular characterization, including the identification of critical pathway changes such as the ones that occur due to *BRAF* mutations, and in finding drugs both capable of passing through the BBB and exceeding the resistance capacity of CNS tumors. Pediatric tumors with *BRAF* mutation partially overlap the spectrum of adult tumors with the same mutation; the *BRAF* V600E mutation in particular can be found in Langerhans Cell Histiocytosis (LCH), papillary thyroid carcinoma and melanoma.

In low-grade gliomas, adults have many mutations in genes such as IDH1 and TP53, but almost never in *BRAF*, while in pediatric tumors, about 85% have one of the two most commonly found abnormalities in *BRAF* (*BRAF* V600E mutation and *BRAF*-KIAA 1549 fusion) and are potential candidates for treatments with drugs inhibiting this pathway. The study of the RAS/RAF/MEK/ERK pathway in children with brain tumors and neurofibromatosis type I (NF-1), where the germline mutation affects *NF1*, a tumor suppressor gene that acts as a negative regulator for RAS, showed how a defect in the activation of RAS may determine the development of low-grade glioma. The recent discovery that most low-grade sporadic pediatric gliomas have *BRAF* mutations has focused efforts on understanding biology related to this biochemical pathway and its pathological activation [6,7].

In this review, we investigate the main aspects and functions of MAPK and PI3K/AKT/mTOR pathways, with a focus on the trials about *BRAF* and MEK targeted therapies available for CNS pediatric tumors, as summarized in Table S1.

## 2. Blood-Brain Barrier (BBB) and Brain Drug Delivery

The research for new targeted therapies needs to address the issue of actual delivery of the drug to the CNS. The most selective physical barrier through which the brain interfaces with the external environment is the BBB. The BBB separates the lumen of blood vessels from the brain, through junctions in the endothelial cells of the blood vessels that limit paracellular passage of external substances. Many misconceptions about the BBB still exist and are variously addressed in the literature, first of all drug distribution into the CSF is not a measure of BBB permeability but rather shows how much of the drug is transported across the choroid plexus. Another important misconception is that CSF is capable of distributing a drug into brain tissue, instead of the drug being injected into the CSF, which tends to mean it is distributed preferentially into the blood, only affecting the ependymal surface of the brain and the spinal cord and not the deeper part of the CNS [8]. The BBB doesn’t only act a simple physical barrier, but also a selective interface which transports molecules into the CNS through various mechanisms, like facilitated diffusion and active transport. Drugs with molecular weight of <500 Dalton (Da) and high lipophilicity are eligible for system delivery (via the blood stream). Other molecules that are not small or lipophilic enough have been the object of study to find a solution that can guarantee their systemic delivery; some of the new techniques employed to deliver such molecules through the BBB are the use of liposomes for making them lipophilic and the use of nanomedicine techniques [9,10]. On the other hand, the techniques that can disrupt or circumvent the BBB have been studied, such as microbubble-mediated ultrasound, intranasal delivery, and intra-arterial delivery without definitive evidence of enhanced mortality [11,12]. MAPK pathway inhibitors have been the object of ample study in melanoma brain metastases. In fact, many small molecules are subject to active efflux from the BBB. Vemurafenib has been proven to be a substrate for P-gp (P-glycoprotein), one of the most extensively studied ATP-binding cassette (ABC) transporters, that plays an active role in the efflux of drugs from CNS. Trametinib has been shown to poorly cross intact BBB [13]. BBB disruption in primary brain tumors might be different from the vascular model studied in secondary tumors like melanoma brain metastases and should be the object of separate studies. In fact, to the best of our knowledge, there is no active study regarding brain drug delivery of *BRAF* and MEK inhibitors in primary pediatric brain tumors. Multimodal drug delivery approaches should be explored, and further in vivo studies are needed to determine actual level of free drug in the brain [14]. A better understanding of the actual drug delivery to the CNS, for both new molecules and drugs that have already proven their efficacy by targeting the MAPK pathway and are orally administered, might help reduce dose-related toxicities while still effectively treating the disease.

## 3. *BRAF* Function and Pathologic Activation

Studies on mammalian cells have seen how the MAPK pathways transmit, amplify, and integrate signals by different extracellular stimuli that are involved in cell proliferation and differentiation but also in the inflammatory response and apoptosis [15].

**Figure 1:**
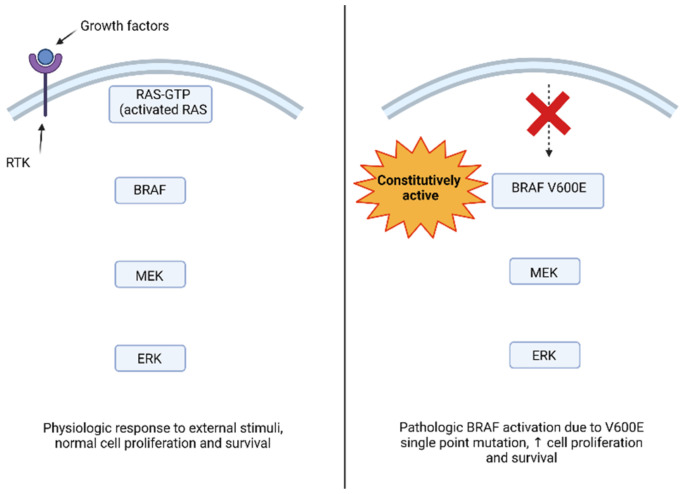
A single point mutation causes the change from valine to glutamine, leading to constitutive activation of BRAF and its downstream effectors.

Cell proliferation is a complex process, regulated by extracellular growth factors, which activate intracellular cascades through the MAPK pathway. These transduction pathways transmit signals through sequential activation of three or five layers of protein kinases, identified as MAPK kinase kinases (MAP4K), MAPK kinase kinase kinase (MAP3K), MAPK kinases (MAPKK or MEK), and MAPK and MAPK activated protein kinases (MAPKAPK) [16]. Four MAPK cascades have been defined based on the components in the MAPK layer: ERK1/2, c-Jun N-terminal kinase (JNK), p38 MAPK, and ERK5 [17]. The ERK1/2 cascade was the first one to be described and is considered as the prototype of these kinase cascades. The activation of extracellular receptors stimulates the activation of GTPase RAS on the plasma membrane, which in turn activates the downstream signaling pathways. Activated RAS recruits the MAP3K level of the cascade (mainly Raf-1, B-Raf, Rafs) in the plasma membrane to induce their activation [18]. Subsequently, the signal is propagated to MAPKKs, MEK1, and MEK2 (MEK1/2) [19] and, to a lesser extent, to the alternative spliced form MEK1b by phosphorylation of two Ser residues in their activation cycle. MEK1/2 are activated and transmit their signal to ERK 1 and ERK2 (ERK1/2), and ERK1b and ERK1c alternately splice into the MAPK layer, causing phosphorylation of the Thr and Tyr regulatory residues in the Thr-Glu-Tyr domain within their activation cycle. Finally, the signals continue to the MAPKAPK components, p90 ribosomal S6 kinase (RSK), the MAPK-interacting kinase (MNK), the mitogen- and stress-activated kinase (MSK), and many other substrates that are located in the cytoplasm, nucleus, or other cellular organelles [20]. The *BRAF* gene is localized on chromosome 7 (7q34) and encodes BRAF protein, a serine/threonine-protein kinase whose action takes place in the RAS-RAF-MEK-ERK signaling pathway, which is an intracellular signal transducer that responds to the stimulus of extracellular growth after acting on specific transmembrane receptors. Pathway activation begins with RAS-GTP assimilation with the RAS domain located within the N-terminal regulatory region of the RAF kinase, leading to the activation of three proteins (ARAF, BRAF, and CRAF/c-RAF-1). The binding of RAS with RAF causes recruitment of the RAF proteins to the cell membrane and RAF activation by conformation changes. As a result, the RAF activates MEK and ERK by phosphorylation, which in turn activates the transcription factors downstream to Elk-1, c-Fos, and c-Myc, which act on cell growth, differentiation, proliferation, and apoptosis. For this reason an aberrant signaling of the MAPK pathway can lead to tumorigenesis [21,22,23]. BRAF is an activator of MEK1/2 with subsequent activation of ERK1/2 (see Figure 1 for a visual representation of the MAPK and PI3K/AKT/mTOR pathways and relative targeted therapies). Growth factors, like the epidermal growth factor (EGF), one of the first growth factors studied about the activation of RAS, manage to activate the pathway by binding to receptors that have tyrosine kinase activity and are thus defined as receptor tyrosine kinases (RTKs). Downstream activation of the MAPK pathway by growth factors allows activated ERK to enter the nucleus of the cell and phosphorylate transcription factors like c-Fos and c-Myc [24].

Genomic analysis of human cancers detected *BRAF* mutations in a high portion of various solid tumors, including brain tumors. The majority of *BRAF* mutations are located within the exon 11 and 15 kinase domains, affecting residues that normally stabilize the kinase in the inactive form. *BRAF* mutations in these locations provoke increased BRAF kinase activity and constitutive activation of MAPK pathway downstream [25]. The most common single point mutation in human cancers is *BRAF* V600E which results in an amino acid change from valine to glutamic acid, rendering the kinase constitutively active [26].

The mutated BRAF kinase activates downstream components of the pathway in the absence of an external signal, even when switching off the cell proliferation signal or initiating apoptosis may be appropriate or necessary. The result of impaired signaling within the cell leads to an alteration in gene expression leading to the proliferation and survival of unregulated cells, contributing to tumorigenesis [27,28].

*BRAF* mutations have been categorized into a three-class system according to their effect on the activity of BRAF protein, as reported in Table 1. Class 1 mutations are RAS-independent signaling as monomers, class 2 mutations are RAS-independent signaling as dimers, and Class 3 mutations are RAS-dependent with impaired kinase activity.

The most common are Class I mutations, which include mutations exon 15 p.V600. These mutations are hyperactivate kinases, promoting the activation of MEK/ERK regardless of RAS activation and protein dimerization, as shown in Figure 1. Physiological regulation of Raf protein kinase activity is determined by Raf proteins homo- and heterodimerization; downstream activation of ERK causes feedback inhibition on the pathway upstream. Class I mutations, like V600E mutated *BRAF*, have innate high kinase activity as a monomer and their dimerization with Raf proteins has little effect on this function; for this reason, ERK upstream feedback inhibition has no effect on Class I mutation, since even if *BRAF* V600E dimerization remains Ras-dependent and is inhibited by ERK upstream feedback, it can still activate the pathway as a monomer [29,30].

**Figure 2:**
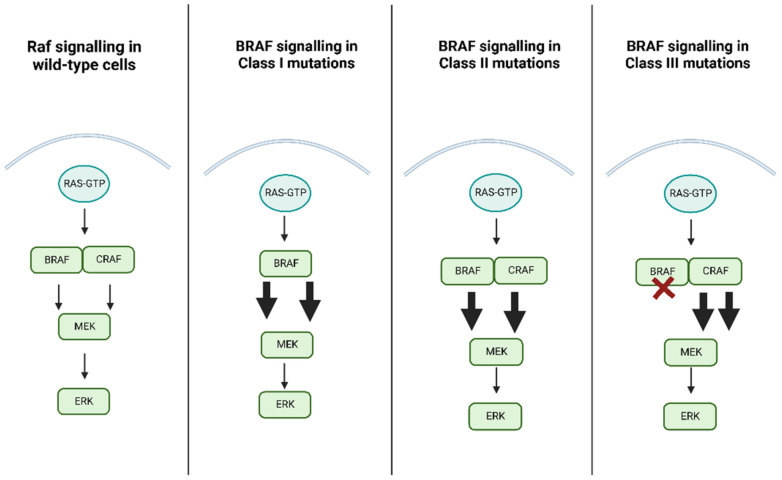
This image shows the physiological regulation and dimerization of *BRAF* and CRAF in wild-type cells. Class I mutations signal as monomers with autonomous kinase activity. Class II mutations signal constitutively as dimers independently of RAS activity. Class II mutations have low or absent kinase activity and are dependent to RAS and need dimerization to activate the pathway. The slim arrows represent physiological kinase activity. The wide arrows represent kinase activity induced by mutations. The red cross symbolizes absent kinase activity as a monomer in Class III mutants.

Class II mutations are less common and involve several point mutations, namely exon 11 p. G464E/V, exon 11 p. G469A/R/V, exon 15 p. L597Q/V, and exon 15 p. K601E/N/T, and also some fusion genes like *BRAF*-KIAA 1549 fusion; it should be noted that this class of mutations is also independent of RAS. Class II triggers both intermediate and high kinase activity. In contrast to class I, class II requires dimerization of the protein to activate the MEK/ERK pathway. Class III mutations, on the other hand, are associated with low or no kinase activity and require both upstream RAS activation and dimerization with CRAF to induce activation of the MER/ERK pathway as shown in Figure 2; in addition, they commonly co-occur with upstream activating alterations such as Neuroblastoma RAS Viral Oncogene Homolog (*NRAS*) mutations, or Neurofibromin 1 (*NF1*) loss, otherwise they require upstream activation by RTK signaling; this determines a high level of RAS in cells harboring this mutation, and for this reason a combination of MEK inhibitor plus an RTK inhibitor could be considered as a possible therapeutic option [31,32,33,34].

RAF presents an ideal target for drug development against cancers. RAF inhibitors, such as sorafenib, vemurafenib, and dabrafenib, have been applied to treat mutated *BRAF* (V600E) tumors, both as single agents and to be administered in combination with MEK inhibitors. Type 1 RAF inhibitor, like dabrafenib and vemurafenib, are ATP competitive molecules able to stabilize RAF in its active conformation while blocking its kinase activity; despite this they induce dimerization of the Raf proteins, bound by the drug, causing a paradoxical ERK activation downstream of the MAPK pathway. Type 2 RAF inhibitors, like sorafenib, stabilize RAF in its inactive conformation and, while they also induce RAF dimerization, they are able to bind to both RAF dimers and inhibit both protomers, reducing their effect on paradoxical ERK activation in wild-type *BRAF* cells; when type 1 RAF inhibitors are bound to RAF, they activate the catalytic domain of the RAF binding partner, leading to an increase in downstream signal, paradoxically activating ERK and can be able to accelerate tumor growth when used alone in patients whose tumors are not driven by Class I mutations and for this reason should not be used in these patients; this same phenomenon has been observed in cells with wild type RAF and is thought to be responsible for these inhibitors’ side effect such as secondary malignancies. When studying a new inhibitor of the MAPK pathway these effects should always be considered and close clinical monitoring for early tumor progression should always be implemented in exploratory studies, especially if no tumor tissue is available for molecular genetic testing [35,36,37,38,39].

## 4. MAPK (Mitogen-Activated Protein Kinases) and mTOR Molecular Pathways

Mitogen-activated protein kinase (MAPK) cascade plays a key role in the transduction of extracellular signals and is a crucial pathway for cancer cell survival, diffusion, and resistance to drug therapy. Aberrant signaling due to the constitutive activation of these pathways can lead to uncontrolled cell growth and survival, starting the tumorigenesis process [40].

Mutations in the RAS-MAPK complex are implicated in several human cancers. Target-based therapies may be the future of cancer treatment and attention has focused on RAS/RAF-MEK-ERK/MAPK signaling pathway inhibitors and its upstream activators. In this context, several MEK1/2 and RAF inhibitors have been clinically tested or are currently being evaluated in the clinical trials [41].

Combined therapy with *BRAF* and MEK inhibitors in *BRAF* V600E mutated tumors showed better activity and reduced toxicity than the use of *BRAF* inhibitors alone. By combining *BRAF* and MEK inhibitors, the *BRAF* signal can be attenuated, while the MEK inhibitor can suppress any non-targeted mutant *BRAF* signal from the targeted *BRAF* agent, simultaneously inhibiting the paradoxical activation resulting from the effect of *BRAF* inhibitors on *BRAF* dimers; nonetheless, as explained in the section regarding *BRAF* class mutations and *BRAF* inhibitors class types, first class *BRAF* inhibitors should not be used in tumors characterized by Class II mutations [42]. MEK inhibitors are active on the MAPK pathway and are divided into two main classes: non-competitive ATP inhibitors and competitive ATP inhibitors. Trametinib (GSK1120212) was the first MEK inhibitor approved by the FDA in May 2013 for the treatment of melanoma. It is an allosteric, non-ATP-competitive inhibitor with activity against MEK1 and MEK2 kinases. The second MEK inhibitor developed was cobimetinib (GDC-0973, XL518), a powerful and highly selective inhibitor, approved by the FDA in November 2015 to be used in combination with vemurafenib for the treatment of unresectable or metastatic melanoma with a *BRAF* V600E or V600K mutation [43]. Other molecules such as the combination of dabrafenib and trametinib, a *BRAF* inhibitor and a MEK inhibitor, respectively, and selumetinib, a highly selective ATP-noncompetitive MEK allosteric inhibitor have also been investigated [44,45]. A phase 2 study that enrolled children with low grade recurrent or refractory glioma (pLGG) found selumetinib to be effective even in patients with type 1 neurofibromatosis (NF-1)-associated pilocytic astrocytomas (PA) both in tumors *BRAF* V600E mutated and tumors that were characterized by *BRAF*-KIAA 1549 fusion [46].

Another pathway that regulates cell replication is mTOR (mammalian target of rapamycin), which links growth factors, nutrients, and energy availability to cell survival, growth, proliferation, and motility [47].

The mTOR pathway responds to growth factors through the phosphatidylinositol 3-kinase pathway (PI3K). The PI3K/AKT/mTOR pathway is activated by transmembrane tyrosine kinase growth factor receptors, such as ErbB family receptors and fibroblast growth factor (FGFR) receptors, the insulin-like growth factor 1 receptor (IGF-1R) and others. PI3K is a kinase activated by multiple mechanisms, such as the binding of growth factors to receptor tyrosine kinases or G-protein-coupled receptors, but also by oncogenes such as RAS. The binding of these ligands to their receptors activates the recruitment of phosphorylation of receptor substrates and subsequently the recruitment of PI3K. PI3K converts phosphatidylinositol-4,5-phosphate (PIP2) in the cell membrane to phosphatidylinositol-3,4,5-phosphate (PIP3). The accumulation of PIP3 is antagonized by lipid phosphatase PTEN. PIP3 recruits phosphoinositide-dependent kinase 1 (PDK1) and Akt to the membrane, resulting in phosphorylation and activation of Akt by PDK1. mTOR is connected to the PI3K pathway by the proteins of tuberous sclerosis TSC1 (hamartin) and TSC2 (tuberin). The tuberin–hamartin complex is a GTPase-activating protein (GAP) towards Rheb (Ras homolog enriched in brain), which causes hydrolysis of GTP by Rheb and conversion from the active GTP-bound form to the inactive GDP-bound form. After activation of the PI3K pathway, phosphorylation of TSC2 by upstream AKT kinase inhibits the TSC1/TSC2 complex and allows mTOR activation by Rheb, thus promoting signal propagation [48,49]. TSC1 and TSC2 form a heterodimer that negatively regulates the mTOR signaling [50]. Two functional complexes of mTOR can be described: complex mTORC1 and complex mTORC2. mTOR, raptor, mLST8, and PRAS40 are components of the complex mTORC1, which is extremely sensitive to rapamycin, which is the target of first-generation mTOR inhibitors [51]. mTOR, Rictor, Sin1 and mLST8 form the mTORC2 complex which is less sensitive to rapamycin and is known to activate Akt, thus promoting cell proliferation and survival, however its role in normal cell function and tumorigenesis has not yet been completely clarified [52]. Within the cells there is an interconnection between mTOR and MAPK, since mTOR is also activated by mitogenic signals that are transmitted via RAS/MEK/ERK. ERK and RSK (ribosomal S6 kinase) are able to induce mTORC1 activation by inducing inhibitory phosphorylation of TSC2; RSK is also able to target the mTORC1 complex directly promoting the complex kinase activity. In addition, the mTOR pathway is also involved in the detection of the energy state of the cell, so the energy depletion conditions within the cell activating AMPK which communicates with mTOR either by direct or indirect inhibition of mTORC1 [53]. AMPK (adenosine monophosphate-activated protein kinase) inhibits mTOR indirectly by phosphorylating the tumor suppressor TSC2 [54]. mTOR activity is often upregulated in human cancer. Aberrant activation of mTOR can be attributed to different mutations activating the mTOR pathway such as amplification or overexpression of components of mTOR complexes and also mutations or loss of function of negative mTOR regulators [55]. Activation of PI3K facilitates both mTORC1 and mTORC2 activation. Mutations in KRAS and *BRAF* can lead to activation of mTORC1 [56]. Since mTOR plays an important role in tumor progression, mTOR inhibitors are also studied in targeted cancer therapy. Activation of mTORC1 downstream of PI3K and AKT supports cell survival, growth, and proliferation. mTORC2 also increases cell proliferation and survival through the regulation of protein kinases, including AKT, which overall provides significant motivation for additional studies on therapeutic targeting of mTOR complexes in cancer [57]. PI3K-AKT-mTOR pathway studies have led to the development of several distinct classes of drugs, including PI3K and AKT inhibitors, as well as allosteric mTOR and mTOR kinase inhibitors [58].

Most cancers driven by PI3K/AKT/mTOR signaling aberrations are marked by PI3K kinase mutations. As an example tuberous sclerosis complex (TSC), an autosomal dominant genetic disorder, is characterized by mutations in TSC1 or TSC2 resulting in an inappropriate mTORC1 signaling which is thought to be responsible for the manifestation of this condition, like renal angiomyolipomas (AMLs) and subependymal giant astrocytoma (SEGA); mTOR inhibitor everolimus has been used for the treatment of both adult and children with AMLs and SEGA according to the data from the clinical trials EXIST-1 and EXIST-2. Many mTOR allosteric inhibitors have been developed for target therapy in clinical oncology (Everolimus, Temosirolimus, and Sirolimus), mTOR kinase inhibitors (Vistusertib), Pan-PI3K inhibitors (Copanlisib, Buparlisib, Pilaralisib), PI3K-alpha inhibitors (Alpelisib, Taselisib), and AKT inhibitors (Ipatasertib) [59,60].

**Figure 3:**
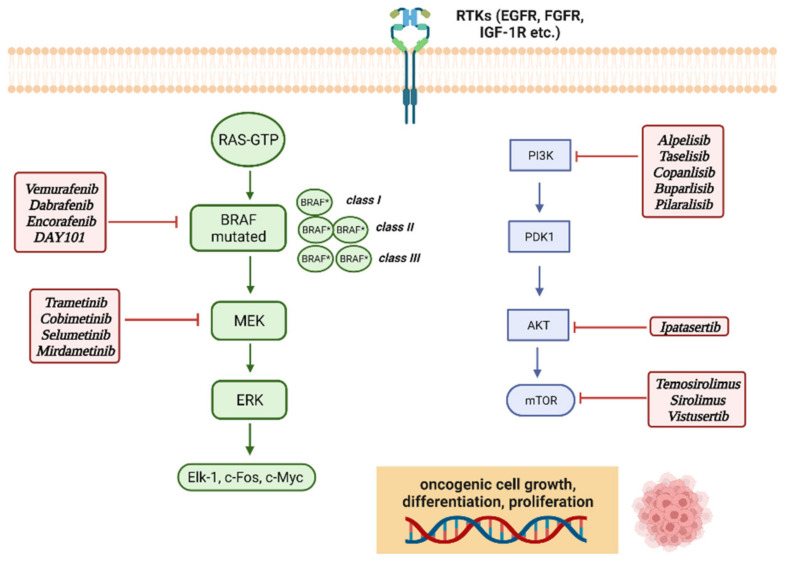
RTKs: receptor tyrosine kinases; EGFR: epidermal growth factor receptor; FGFR: fibroblast growth factor receptor; IGF-R1: insulin-like growth factor 1 receptor; RAS: Rat sarcoma (reflecting how the first member of the RAS gene family was discovered); *BRAF*: v-raf murine sarcoma viral oncogene homolog B1; MEK: mitogen-activated protein kinase; ERK: extracellular signal-regulated kinase; Elk-1: ETS (erythroblast transformation specific) like-1 protein; c-Myc: c-myelocytomatosis oncogene product; PI3K: phosphatidylinositol-3 kinase; PDK1: 3-phosphoinositide-dependent kinase 1; mTOR: mammalian target of rapamycin: BRAF*: mutated *BRAF*.

Figure 3 overviews targeted therapies currently available toward mutant MAPK and PI3K/AKT/mTOR pathways.

## 5. *BRAF* and MEK Inhibitors in Pediatric CNS Tumors

### 5.1. Pediatric Low-Grade Glioma (pLLG)

Pilocytic astrocytoma (PA) is the most common pediatric low-grade glioma (pLGG), accounting for approximately 15.6% of brain tumors that arise up to 19 years old [61]. Its development is more common in the first two decades of life, with peak of incidence from age 0 to 9. PAs are most commonly found in infratentorial structures such as the cerebellum but they also frequently arise in midline brain structures like the optic nerves, the hypothalamus, and the brain stem. The updated WHO 2021 classification describes this entity as a grade 1 lesion, due to its slow potential for growth, which correlates to its good prognosis with a 10-year overall survival estimated between 85% and 96% [62]. Nevertheless, PA may impact heavily on the child’s quality of life with frequent neurological and endocrine complications due to the lesion itself or to the prolonged treatment needed. The mainstay of therapy for symptomatic or progressive PA is complete surgical resection whenever surgically feasible. If the tumor is completely resected, no further therapy is needed; incomplete resection, most frequently due to the anatomical location, can be followed by a prolonged period of stability of the lesion, nonetheless, some cases may require additional therapy. In the absence of radical surgery, chemotherapy or radiotherapy may be adopted to treat the residual lesions, so the main focus is still on optimizing long-term treatment to reduce early and late side effects. The most commonly used chemotherapy regimen is carboplatin and vincristine for intensified induction chemotherapy, or vinblastine alone [63].

PA may not completely respond to first-line therapy, requiring extensive radiological and clinical monitoring to accurately determine the need for additional therapy. Radiotherapy is to be employed in progressive and refractory disease only and it should be avoided in younger children because of long-term adverse effects. Moreover, at the molecular level, PA often shows alterations in MAPK pathways, mostly point mutations in genes and fusions which include genes such as *BRAF*, KRAS, FGFR1, *NF1*, and many more. MAP/ERK pathway mutations are found in more than 90% of PA; the most commonly found genetic aberration is the KIAA1549-*BRAF* fusion in 60–70% of cases, while *BRAF* V600E mutation is found in 10% of PA [64].

Numerous agents targeting the MAPK pathway, such as MAP/ERK kinase or *BRAF* inhibitor, are currently being tested. One of the most extensively studied drugs is selumetinib (AZD6244); a phase 2 trial (NCT01089101) enrolled 25 eligible patients for treatment with selumetinib at the recommended dosage of 25 mg/sqm/dose bis in die (BID) for a maximum of 26 courses. Out of these 25 patients, six (6/25, 24%) showed partial response, fourteen (14/25, 56%) had stable disease while five patients had a progression of disease. Two-year progression-free survival (PFS) was 78% ± 8.5%. Nineteen patients out of the 25 who were enrolled underwent visual acuity evaluation: five of them showed improvement of the visual fields while the other fourteen patients showed stability of the visual fields. Among the most common toxicities were grade 1 and 2 creatine phosphokinase elevation (CPK), aspartate aminotransferase (AST), and alanine aminotransferase (ALT) elevation, hypoalbuminemia, skin rash, vomiting, diarrhea, headache, and anemia. These results show that selumetinib may be well tolerated and can be effective to prolong disease stability in children with recurrent or progressive optic pathways and hypothalamic glioma. All of the 25 patients had received chemotherapy previously and 19 of the 25 received surgery before treatment with selumetinib [65]. A phase 3 clinical trial (NCT04166409) compares carboplatin and vincristine with selumetinib alone in previously untreated pLGG, that do not have a *BRAF* V600E mutation and are not associated with systemic neurofibromatosis type 1 [66].

Trametinib is another MEK-1/2 inhibitor, studied in the pediatric trial NCT02124772 both alone and combined with dabrafenib, a *BRAF* inhibitor. Route of administration is a crucial factor in pediatric patients. Younger children might not be able to swallow bigger pills, capsules or tablets and the dosage offered by solid forms of the drugs might be inadequate for them. In the trial, both trametinib and dabrafenib were available in tablets as well as oral suspension. This trial also accounted for the palatability of the oral suspension together with how easy they were to reconstitute and administer to the patients. Adherence to therapy is always a factor to be considered in pediatric patients and oral suspensions are useful both for patients who cannot properly swallow tablets and for measuring in an easier way the correct dosage. Preliminary available data show that the oral solutions of trametinib and dabrafenib were not difficult to swallow for the patients, but no clear conclusion can be drawn on palatability because of missing data collected from patients over 12 years old. In Part D of this trial a separate section was designed for participants affected by pLGG to be treated with trametinib in combination with dabrafenib (see Table 1 for dosage used). Maximum observed plasma concentration (Cmax) of dabrafenib in this group was 1360 ng/mL, it should be noted that this geometric mean was obtained on an analysis on only nineteen of the twenty patients enrolled in this group. Of the twenty patients, eight suffered from serious adverse effect (8/20, 40%) ranging from decreased white blood cell count to seizures (only one event reported) and decrease in cardiac ejection fraction (only one event reported); there was also one episode of tonsillitis and one episode of respiratory distress [67].

There are many MEK inhibitors (like cobimetinib and binimetinib) and many *BRAF* inhibitors (like vemurafenib and dabrafenib) which are currently being studied and tested as possible targeted therapies. A completed phase I/II clinical trial (NCT01677741) enrolled 32 patients with recurrent, progressive or refractory solid tumors with *BRAF* V600E mutation with the aim to study the safety, tolerability, and pharmacokinetics of dabrafenib. Dabrafenib was available both as capsules and as oral suspension; the latter was to be used for any patient unable to safely swallow capsules. The data regarding pLGG enrolled in the trial showed a response rate of 44% and a 1-year estimated PFS of 85% [68].

A pediatric phase 2 trial that aimed to test sorafenib in children with recurrent LGG was halted when unexpected progression happened in 9 of 11 patients, 3 of which had the KIAA1549-*BRAF* fusion and *NF1*. This effect has been confirmed to be due to paradoxical extracellular signal-regulated kinase (ERK) activation, which was demonstrated in vitro both in *BRAF* wild type, *BRAF* fusion, and *NF1*-deficient tumor cells in vitro [69]. It was also proven that KIAA1549-*BRAF* fusion kinase functions as a homodimer that is resistant to the first generation of *BRAF* inhibitors, like vemurafenib, which targets the monomeric form of *BRAF*. For this reason, first generation *BRAF* inhibitors should not be used in glioma which shows this fusion. Second generation *BRAF* inhibitors, like DAY101 (formerly TAK-580, MLN2480), are able to target both monomeric and dimeric forms of *BRAF* bypassing the paradoxical activation of the pathway. In the trial NCT03429803 pediatric patients with radiological evidence of recurrence or progression of disease of non-hematologic malignancies that had evidence of activation of the MAPK pathway were treated with DAY101, aiming for the maximum tolerated dose (MTD) [70].

Pleomorphic xanthoastrocytoma (PXA) is a rare primary CNS tumor most commonly diagnosed in the second decade of life, without any gender predilection, with cases as young as 2 years reported [71]. Like other primary CNS tumors, molecular characterization is important for the prognosis at the moment of the diagnosis, but the WHO classification system has not yet officially described PXA *BRAF* mutated and *BRAF* wild-type as distinct clinical entities. The 2021 WHO classification of CNS tumors defines PXA a grade 3 lesion only if it shows by ≥5 mitoses per 10 HPF (high power field); if it has less it is considered a grade 2 lesion. The most common primary location is the temporal lobe [72]. The most frequently mutated gene in PXAs is *BRAF*, which can be found in 2/3 of typical PXA, less commonly in its anaplastic variant, which might imply different molecular pathogenesis. *BRAF* V600E mutation is the one more commonly observed, but fusions of *BRAF* and other different mutations have been described [72,73]. OS and PFS are worse in the anaplastic PXA, but the presence of *BRAF* V600 mutations is associated with longer OS rates both in typical and anaplastic PXA. Due to the rarity of PXA, optimal management of these lesions must take into consideration case reports and case series. Gross total resection was associated with longer PFS, but not with better OS, if compared with subtotal resection and biopsy (5-years PFS 92.3% vs. 41.7%, *p* = 0.0002) [74]. Similarly, radiotherapy may play a role in residual or recurrent disease. The role of systemic therapy is still being defined but it should be noted that as highlighted in a recent brief review of literature from 2019 regarding PXAs by Shaikh et al., traditional chemotherapy is considered minimally effective in the treatment of PXAs. Given the high incidence in PXA of targetable mutations there have been clinical trials ongoing regarding the use of *BRAF* inhibitors both in monotherapy and associated with MEK inhibitors.

A phase 2 clinical trial (NCT05180825), called PLGG-MEKTRIC, is ongoing for comparing trametinib (Mekinist™) with standard chemotherapy with vinblastine during 18 courses of 4 weeks each in pediatric low-grade glioma and mixed glioneuronal tumors, including PXA, without *BRAF* V600E mutation or correlation to NF-1. Its primary endpoint is 3-year PFS, but data regarding the difference in PFS and OS according to molecular biomarkers are also analyzed [75].

Another phase 2 clinical trial (NCT02684058) will investigate the activity of dabrafenib in combination with trametinib in two different cohorts, LGG and high-grade glioma (HGG) with *BRAF* V600E mutation, actively comparing the LGG experimental cohort with traditional chemotherapy with carboplatin and vincristine. The primary endpoint is the overall response rate (ORR) in the first 32 weeks of treatment. ORR will be assessed through MRI and/or CT scans using Response Assessment in Neuro-Oncology Criteria (RANO) criteria [76]. The results from the VE-BASKET study which is an open-label, non-randomized, multicohort study for *BRAF* V600E-mutant non melanoma tumors, showed that in seven PXA treated with vemurafenib only one showed complete response, two showed partial responses, and three patients had stable disease. In this study, arthralgia, melanocytic nevus, palmar-plantar erythrodysesthesia, and photosensitivity reaction were the most common adverse effect, whereas maculopapular rash was the most common grade 3 and 4 event and no grade 5 treatment-related events were observed. These results confirmed that vemurafenib shows safe antitumor activity in some patients with *BRAF* V600E mutant glioma, with the highest response rate observed in low-grade tumors, such as PXA [77].

Oligodendroglioma and diffuse astrocytoma were originally part of a broad group, which generically described them as diffuse gliomas. The tumors that showed histological characteristics common to both types of lesions were also included in this group. The 2016 WHO classification of Tumors of the Central Nervous System changed this by introducing a differentiation on a molecular basis. As an example, the diagnosis of anaplastic oligodendroglioma requires the presence of two mutations: both isocitrate dehydrogenase 1 or 2 mutations (IDH-mt) and 1p/19q co-deletion have to be present. Anaplastic astrocytoma on the other hand was divided into IDH wild type and IDH mutated tumors. IDH-mt tumors usually present themselves with low-grade histology at the diagnosis that tends to evolve slowly in time, nonetheless they have a more favorable prognosis than IDH-wt tumors. Diffuse astrocytoma and oligodendrogliomas account for 13% of primary brain and other CNS gliomas [78]. Oligodendrogliomas in pediatric and young adult patients are rare and their molecular pathogenesis has been shown to be different from that of oligodendroglioma in adults [62]. A study reported two cases of grade II and grade III oligodendroglioma respectively in a 14-year-old girl and an 11-year-old boy. In both cases, no evidence of 1p/19q co-deletions or mutations of IDH1, TP53, CIC, and H3F3A genes were found. Instead, both cases showed MAPK/ERK pathways activation as proven through immunohistochemical analysis and RT-PCR analysis and Sanger sequencing that showed the presence of KIAA1549_Ex15-*BRAF*_Ex9 fusion protein. This was the first study that demonstrates the occurrence of KIAA1549-*BRAF* fusion in pediatric oligodendroglioma, highlighting the importance of molecular characterization at the diagnosis. Further longitudinal studies are required to better describe the incidence of these mutations as a possible target for therapy [79]. It should be noted that pediatric diffuse gliomas rarely have the above-mentioned genetic mutations. The rate of *BRAF* mutation in the pediatric diffuse glioma is around 3% for fusion and 8%–43% for V600E, implying that pediatric diffuse glioma has a different molecular underpinning from the diffuse glioma that manifests in the adult [63,80]. As it has become the norm for other low and intermediate lesions, it has become common practice to operate early on low-grade glioma-like lesions when radical surgery is considered feasible and safe. As for PAs and PXAs, focal radiation therapy is a possible approach for unresectable or recurrent diseases but should be reserved for more aggressive lesions because of the long-term side effects, especially on cognitive development, that are more severe if radiation therapy is used in the first years of life [81]. Currently, new radiation techniques may be adopted, like proton therapy that can minimize the damage to adjacent structures. The trial NCT04065776 is currently ongoing to determine the feasibility of hippocampal avoidance (HA) for pLGG located in the midline or suprasellar region, and the clinical outcome is being assessed comparing various neurocognitive scores, which mainly focus on memory as a direct measure of hippocampal damage. Depending on tumor location, the dosage used will be 52.2 CGE or 54 CGE in 29 or 30 fractions. To the best of our knowledge there is no trial that has compared chemotherapy alone with chemotherapy and radiotherapy combined; because of this combination therapy might be considered in relapsed or recurrent disease after first line treatment with the age of the patients being one of the most important factors in deciding whether or not radiation therapy should be implemented [80]. The trial NCT04923126 is an open-label, multi-center, phase ½ study of the MEK inhibitor mirdametinib (PD-0325901), which preclinical studies have reported to have potentially superior blood-brain-barrier penetration compared to other MEK inhibitors [12], in patients with pLGG. Both patients with relapsed or progressed disease and previously untreated subjects are eligible for the study, in the presence of MAPK pathway activation or *NF1*, NF2, and other germline mutations. The treatment with mirdametinib in this trial is going to be administered twice daily on days 1–28 for up to 26 cycles (24 months) in the absence of disease progression or unacceptable toxicity. Interim results as recently reported by Vinitsky et al. are promising; of eleven patients recruited six had at least one follow-up disease evaluation: four of them showed minor response (>25–50% decrease); no disease progression has been observed; there were no grade 3 or 4 adverse events; and no MEK-related cardiomyopathy or retinopathy [82].

### 5.2. Pediatric High-Grade Glioma (pHGG)

Pediatric High-Grade Glioma (pHGG) comprises almost 15% of all primary brain tumors in children. pHGGs encompass many clinical entities that are very different from each other, for their histological and molecular features. Molecular profiling of pediatric HGGs is different from HGGs of the adult [83]. Different histological subtypes of pHGGs can harbor distinct genetic drivers that can offer potential therapeutic targets and offer a better prognosis, like *BRAF* mutations in epithelioid glioblastoma and anaplastic pleomorphic xanthoastrocytoma (aPXA) [84].

The 2021 WHO classification of Tumors of the Central Nervous System distinguishes four types of diffuse pediatric high-grade gliomas: diffuse midline glioma H3 K27-altered; diffuse hemispheric glioma H3 G34-mutant; diffuse pediatric-type high-grade glioma H3-wildtype; and IDH-wildtype, infant-type hemispheric glioma [85]. However, most articles cited in this paper are dated before the 2021 WHO classification, consequently in this section, we are going to discuss hemispheric pHGGs, like anaplastic astrocytoma, glioblastoma (GBM), and high-grade midline tumors, formerly diffuse midline glioma (DMG), in order to avoid confusion.

Anaplastic astrocytoma (incidence 0.1/100.000 patients from age 0 to 19 years) is a Grade 3 lesion and GMB (incidence 0.18/100.000 patients from age 0 to 19 years) is a Grade 4 lesion, these are the clinical entities that constitute hemispheric HGG [61]. Gliomatosis cerebri (GC) is not considered a distinct clinical entity since the 2016 WHO classification; instead, it can be described as a highly infiltrative growth pattern that can be considered a phenotypic manifestation of HGG, both in pediatric patients and adults [86,87]. Although there is no widely accepted recommended standard of care, and treatment must be tailored for each patient, most surgically approachable lesions undergo gross tumor resection (GTR) followed by focal irradiation and additional chemotherapy, most commonly temozolomide, an oral alkylating agent, usually both during and after radiation therapy. A better understanding of the tumor’s molecular background is a possible step toward increasing survival. A 20-year systematic review and meta-analysis of 129 patients in 2018 showed a cumulative OS of 4.0 months (95% CI 1.9–6.1) [88]. The incidence of *BRAF* mutations in adult GBM is estimated to be 1–3% while in teenage patients and young adults GBM these mutations are much more frequent with incidence up to 50% in the epithelioid variant [89]. The revised 2016 WHO classification of tumors of the CNS was the first one to introduce a new clinical entity: the diffuse midline glioma (DMG) H3 K27M mutated, designating it as a distinct entity from other midline lesions. The presence of H3 K27M alterations in any infiltrating midline gliomas determines the assignment to WHO grade IV. H3 K27M alterations may rarely occur in low-grade midline gliomas and posterior fossa ependymomas, but the clinical relevance of this occurrence is not yet fully understood [90]. Pagès et al. in 2018 reported a co-occurrence of H3 K27M and *BRAF* V600E mutation in five pediatric midline gangliogliomas; all five cases were Grade 1 without anaplastic features and one of them underwent spontaneous malignant in situ transformation 7 years after the diagnosis. The results of the data from this report suggested that the presence of H3 K27M mutation in tumor with no malignant feature should not automatically define the lesion as Grade 4 and that *BRAF* status should always be assessed. There are only a few cases reported in literature of these two mutations occurring simultaneously and their meaning is not fully understood and should be investigated further. High-grade midline gliomas, similarly to hemispheric pHGG, have an unfavorable prognosis, with a median survival time of less than 1 year. Commonly only a stereotactic biopsy is performed since most of these lesions show diffuse growth patterns making them ineligible for radical surgery [91].

There are numerous ongoing clinical trials regarding targeted therapies toward *BRAF* and MAPK pathways among pediatric HGGs. The trial NCT03919071 is actively recruiting pediatric patients with newly diagnosed HGG with *BRAF* V600E mutations and without H3 K27M mutation in order to treat these patients firstly with radiation therapy and then with a combined therapy comprised of dabrafenib and trametinib in order to estimate the event-free survival (EFS) to compare this EFS to contemporary historical controls. Therapy with dabrafenib and trametinib is going to be administered four weeks after completion of RT. The patients will receive dabrafenib mesylate orally twice daily and trametinib once daily on days 1–28. In this trial, treatment repeats every 28 days for up to 24 cycles in the absence of disease progression or unacceptable toxicity [92]. The trial NCT03220035 is a phase II of the pediatric MATCH trial, which aims to study how effective vemurafenib is in treating patients with tumors with V600E mutations that have advanced locally, have relapsed, recurred, or do not respond to treatment, with the primary objective of determining the response rate. In this trial patients will receive vemurafenib orally on days 1–28. Like other trials, cycles of therapy repeat every 28 days in this case for up to 2 years in the absence of disease progression or unacceptable toxicity [93]. Other trials like NCT02639546, currently completed, have tried to evaluate the safety, tolerability and pharmacokinetics of newer molecules, such as cobimetinib, with a dose-escalation stage and an expansion stage after finding the recommended dose. Cobimetinib is available and has been used in this trial, both in tablet and suspension form. Only five cases of HGG were recruited and none of them showed complete or partial response after 2 months of therapy. Further studies are still needed to understand which molecular pathway may offer the best results in terms of OS and PFS, but currently, the biggest obstacle is the insufficient number of patients studied due to the low incidence of these tumors [94].

### 5.3. Other Tumors

Ganglioglioma is a rare, slow-growing, and defined tumor, with both cystic and solid neuronal and glial elements, that usually occurs at the pediatric and young adult age. They are considered indolent tumors and surgical resections are potentially curative, however complete resection is not always possible. *BRAF* V600E and *BRAF* fusions have been reported among patients with ganglioglioma [56]. Dayiha et al. studied a large cohort of 53 pediatric patients with ganglioglioma and found that *BRAF* V600E mutation correlates with shorter recurrence-free survival, alerting to the need for the identification of this high-risk group and determining future *BRAF*-targeted therapies and disease surveillance strategies [94].

Diffuse Leptomeningeal Glioneuronal Tumor (DLGNT) is a rare tumor that usually occurs in children and adolescents, characterized by the leptomeningeal spread of oligodendroglial-like cells. Most DLGNT are indolent, but sometimes they can progressively enlarge in size and increase in number, going into an advanced stage [95]. The hallmark molecular feature of this tumor seems to be co-deletion of 1p/19q and the pathologic activation of the MAPK, which may occur in 80% of DLGNT, mostly KIAA1549:*BRAF* fusions, that were found in 66% of them. Thus, MEK inhibitors may be promising therapeutic targets for improving the clinical outcome of patients with DLGNT [96].

Polymorphous Low-Grade Neuroepithelial Tumor of the Young (PLNTY) was described in 2017 by Huse et al. as a new entity of low-grade, oligodendroglioma-like neuroepithelial tumor, with astrocytic and ependymal appearance. The most common location of PLNTY is subcortical in the temporal lobe and because of this, they are frequently epileptogenic tumors. Over-activation of the MAPK pathway is frequently observed in these tumors, making it a potential target for therapy. In Huse’s original series, three of seven cases were *BRAF* V600E mutant and the remaining cases exhibited fusion events involving FGFR2/FGFR3 [97].

## 6. Conclusions

As we improve our knowledge of pediatric CNS tumors, the *BRAF* pathways are receiving growing attention from the scientific community. Since many different alterations of the *BRAF* pathways have been described, it is safe to assume that different mutations will require different and specific classes of inhibitors. Further studies regarding the effect of inhibitors in vivo are needed to this end. *BRAF* and MEK inhibitors may, in the future, significantly reduce the need for classic chemotherapy and radiation therapy in treating pediatric CNS tumors. More clinical trials regarding these promising new molecules are needed among the pediatric population, in order to determine the adequate dosage, duration of therapy, and long-term side effects. Long-term follow-up should always be planned not only to quickly diagnose secondary malignancies, mostly skin tumors induced by MAPK paradoxical activation which have already been described in the literature, but also for new possible long-term side effects and iatrogenic tumors, which have not yet been linked to these new molecules.

## Figures and Tables

**Table 1:** *BRAF* mutations classes.

Class I	Class II	Class III
p.V600D/E/K/M/R	p.G464E/V; p. G469A/R/V; p. L597Q/V; p.K601E/N/T; gene fusion	p.D287H; p.V459L; p.G466A/E/V; p.S467L; p.G469E; p.N581I/S; p.D594A/G/H/N; p.F595L; p.G596D/R

## Supplementary Materials

The following supporting information can be downloaded at: www.mdpi.com/article/10.3390/cancers14174264/s1, Table S1: Clinical trials cited in the paper, Available online: https://clinicaltrials.gov/ct2/home (accessed on 6 June 2022).
