# Supplementary material for: BRAF and MEK Targeted Therapies in Pediatric Central Nervous System Tumors
Source: Cancers (Basel). 2022 Aug 31;14(17):4264. doi: 10.3390/cancers14174264 (PMC9454417; doi:10.3390/cancers14174264)
Supplement: Supplementary file 1 [file cancers-14-04264-s001.zip › cancers-1776334-supplementary-done.pdf]

cancers MDPI

Table S1. Clinical trials summarized in the paper, available on https://clinicaltrials.gov/ct2/home.

Official Title	ClinicalTrials.gov Identifier	Actual Study Start Date	Recruitment Status	Drug or Intervention
A Phase 1 and Phase II and Re-Treatment Study of AZD6244 for Recurrent or Refractory Pediatric Low-Grade Glioma	NCT01089101	April 19, 2010	Active, not recruiting	Selumetinib
A Phase 3 Randomized Non-Inferiority Study of Carboplatin and Vincristine Versus Selumetinib (NSC# 748727) in Newly Diagnosed or Previously Untreated Low-Grade Glioma (LGG) Not Associated With BRAFV600E Mutations or Systemic Neurofibromatosis Type 1 (NF1)	NCT04166409	January 3, 2020	Recruiting	Selumetinib Sulfate
An Open-Label, Dose-Escalation, Phase I/II Study to Investigate the Safety, Pharmacokinetics, Pharmacodynamics and Clinical Activity of the MEK Inhibitor Trametinib in Children and Adolescents Subjects With Cancer or Plexiform Neurofibromas and Trametinib in Combination With Dabrafenib in Children and Adolescents With Cancers Harboring V600 Mutations	NCT02124772	January 15, 2015	Completed	Trametinib Dabrafenib
Phase I/iia, 2-Part, Multi-Center, Single-Arm, Open-Label Study to Determine the Safety, Tolerability and Pharmacokinetics of Oral Dabrafenib in Children and Adolescent Subjects With Advanced BRAF V600-Mutation Positive Solid Tumors	NCT01677741	May 23, 2013	Completed	Dabrafenib
A Phase I Study of DAY101 (Formerly TAK-580, MLN2480) for Children With Low-Grade Gliomas and Other RAS/RAF/MEK/ERK Pathway Activated Tumors	NCT03429803	February 27, 2018	Active, not recruiting	DAY101
A Randomized and Controlled Phase II National Protocol in Non NF1 Pediatric and AYA (Adolescent and Young Adults) Patients Bearing a Wild Type BRAF Gene Newly Diagnosed Comparing a Daily Oral MEK Inhibitor (Trametinib) Versus Weekly Vinblastine During 18 Months	NCT05180825	Estimated start date was February 1, 2022, last update on January 6, 2022	Not yet recruiting	Trametinib
Phase II Open-label Global Study to Evaluate the Effect of Dabrafenib in Combination With Trametinib in Children and Adolescent Patients With BRAF V600 Mutation Positive Low Grade Glioma (LGG) or Relapsed or Refractory High Grade Glioma (HGG)	NCT02684058	December 28, 2017	Active, not recruiting	Dabrafenib Trametinib

SJ901: Phase 1/2 Evaluation of Single Agent Mirdametinib (PD-0325901), a Brain-Penetrant MEK1/2 Inhibitor, for the Treatment of Children, Adolescents, and Young Adults With Low-Grade Glioma	NCT04923126	June 21, 2021	Recruiting	Mirdametinib
A Phase 2 Study of Dabrafenib (NSC# 763760) With Trametinib (NSC# 763093) After Local Irradiation in Newly-Diagnosed BRAF V600-Mutant High-Grade Glioma (HGG)	NCT03919071	October 2, 2019	Recruiting	Dabrafenib Mesylate, Trametinib, Radiation Therapy
NCI-COG Pediatric MATCH (Molecular Analysis for Therapy Choice)- Phase 2 Subprotocol of Vemurafenib in Patients With Tumors Harboring BRAF V600 Mutations	NCT03220035	July 24, 2017	Active, not recruiting	Vemurafenib
A Phase I/II, Multicenter, Open-Label, Dose-Escalation Study of the Safety and Pharmacokinetics of Cobimetinib In Pediatric and Young Adult Patients With Previously Treated Solid Tumors	NCT02639546	May 20, 2016	Completed	Cobimetinib
